# Evaluation of the Effect of Nebulized N-Acetylcysteine on Respiratory Secretions in Mechanically Ventilated Patients: Randomized Clinical Trial

**Published:** 2015-07

**Authors:** Seyed Masoom Masoompour, Amir Anushiravani, Amir Tafaroj Norouz

**Affiliations:** 1 Pulmonary and Critical Care Division, Department of Internal Medicine, Shahid Fahghihi Hospital, Shiraz University of Medical Sciences, Shiraz, Iran;; 2 Department of Internal Medicine, Nemazee Hospital, Student Research Center, Shiraz University of Medical Sciences, Shiraz, Iran

**Keywords:** Acetlycysteine, Randomized clinical trial, Ventilators, Iran

## Abstract

**Background:**

The purpose of our study was to evaluate an inexpensive and available method to reduce mucous impactions in mechanically ventilated patients.

**Methods:**

This randomized clinical trial was conducted on 40 mechanically ventilated patients aged 15-90 years. The patients were randomly allocated into two arms; 20 cases and 20 controls. The cases received N-acetylcysteine via their nebulizers, and the control group received normal saline three times a day for one day. We measured the density of respiratory secretion, plateau and peak airway pressures, and O2 saturation at baseline, 12 and 24 hours later.

**Results:**

Although the mean secretion density was significantly lower in the NAC group (F (1, 38)=8.61, P=0.006), but a repeated measures ANOVA with a Greenhouse-Geisser correction determined that the effect of NAC on mean secretion density did not differ significantly between time points (F (1, 38)=3.08, P=0.087). NAC increased O2 saturation significantly between time points (F (1.92, 73.1)=4.6, P=0.014). The plateau airway pressures were relatively stable throughout the study in the normal saline and NAC groups (F (1.95, 37.1)=0.67, P=0.513). The peak airway pressure did not change significantly during the study in the normal saline and NAC groups (F (1.52, 56.4)=0.91, P=0.384).

**Conclusion:**

Considering the limitations of the study, nebulized NAC in mechanically ventilated patients was not effective more than normal saline nebulization in reducing the density of mucous plugs. The peak and plateau airway pressures were relatively stable throughout the study in both groups.

**Trial Registration Number: **IRCT201104276312N1.

## Introduction

Maintaining airway secretion, in other words airway hygiene, is very important in airway patency and preventing respiratory tract infections. Impaired airway clearance is one of the main reasons for admission to a intensive care unit and can be a cause and/or contributor to acute respiratory failure. Physical methods to intensify airway clearance are often used in the ICU, but only a few are confirmed as effective.^1^

Retained tracheobronchial secretions, usually called mucous plugs, are commonly seen in mechanically ventilated patients due to impaired cough reflex, depressed mucociliary clearance, and increased mucus production.^2^

Shiraz, the capital of Fars province in Iran, has a very dry climate; with a mean humidity of 37.25%, ranging from about 20% in June to 60% in January^3^ which predispose our patients to mucous plugs, causing severe morbidity and even mortality.

Mucolytic therapy has been used to decrease sputum viscosity, increase clearance of sputum, reduce bacterial load, improve lung function, and ultimately increase survival. Anti-DNase medications are very expensive and not available locally, therefore a search for a cheap and effective method to minimize secretion retention, which is a difficult problem to deal with, in mechanically ventilated patients, needs further warrant. N-acetylcysteine (NAC) had been used orally for decades, but its role as a mucolytic agent, administered through the ETT remains unknown. Sheffner first discovered the mucolytic properties of N-acetylcysteine and since then, this drug has been widely employed clinically in situations requiring mucolysis.^4^ N-acetylcysteine acts to reduce mucus viscosity by splitting disulfide bonds linking proteins present in the mucoproteins. After proper administration of NAC, the volume of liquefied secretions may increase. On the other hand, mechanically ventilated patients have inadequate cough, therefore their airways must be maintained open by mechanical suction. Adverse effects such as stomatitis, nausea, vomiting, fever, rhinorrhea, drowsiness, clamminess, chest tightness and bronchoconstriction are seen with the administration of any form of NAC.^5^ Significant bronchospasm is uncommon and unpredictable even in patients with asthmatic bronchitis or bronchitis complicating bronchial asthma.^5^

In a few European countries, N-acetylcysteine has been used regularly to reduce the frequency of exacerbations and improve symptoms in patients with chronic bronchitis. In the UK, the USA, and Australia, mucolytics are used infrequently because they are believed to be ineffective.^6^ The British Thoracic Society guidelines for the management of chronic obstructive pulmonary disease (COPD) claim that there is no role for mucolytics in COPD. These drugs have not been approved by the British National Formulary for use in COPD because clinical trials have not proven their efficacy.^7^ In the USA; only one multicenter controlled trial suggested that organic iodide might have some benefit in the management of chronic bronchitis.^8^

Controlled studies that clearly demonstrate a justification for the use of mucolytics in the ICU setting are not to be found in the literature. This study evaluated changes in secretion density, peak/plateau airway pressures, and oxygen saturation in intubated patients nebulized by NAC in comparison to normal saline nebulization. 

## Materials and Methods

**Figure 1:**
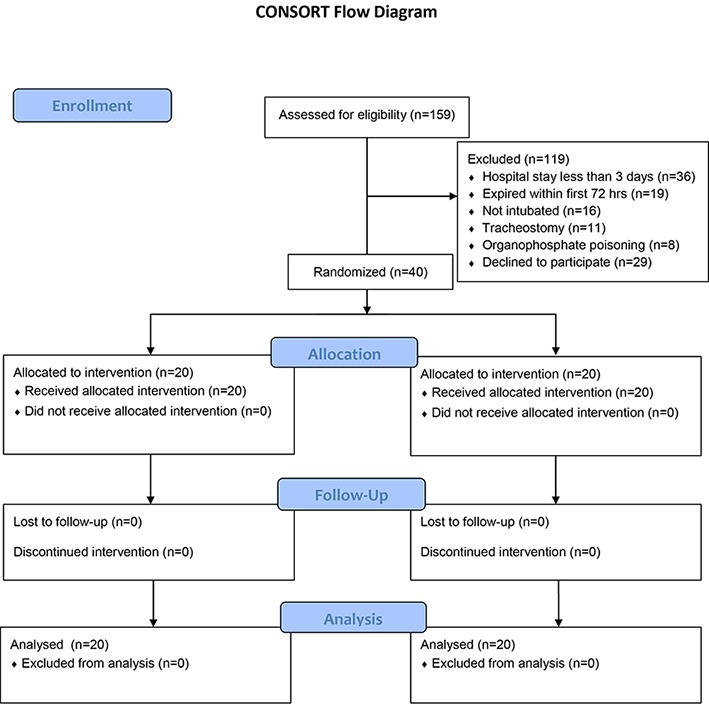
CONSORT diagram shows sampling procedures.

This randomized-controlled clinical trial was conducted in a 10-bed medical ICU at a teaching hospital in Shiraz, from January to the end of June 2012. We studied 40 sedated patients between 15-90 years old, intubated on arrival and mechanically ventilated for more than 72 hours. Patients were randomly allocated into two groups (20 patients per arm) using a computer-guided randomization table (figure 1). For this purpose, 40 cards were placed in a closed box, each having “case” or “control” written on it. Prior to the study, a card was pulled out of the box and a patient was labeled as the case or control.

Exclusion criteria were hemodynamically unstable patients, those with tracheostomy tubes, organophosphate poisonings, and pulmonary edema.

This protocol was approved by the Ethics Committee of Shiraz University of Medical Sciences.

Our ventilators were provided by the Drager Medical Company (Evita 2 and 4 editions). The case group received NAC (Aurum Pharmaceuticas Ltd., UK) via their nebulizers. Prior to our study, we conducted a pilot study using 3 ml of NAC 20% with 3 ml normal saline. This amount of NAC induced bronchospasm, which was relieved immediately using a bronchodilator. Therefore, we decided to reduce the amount of nebulized NAC to 2 ml within 8 ml normal saline. A general practitioner prepared a dilution of 2 ml of NAC 20% with 8 ml normal saline 3 times a day for 1 day. The distinctive sulfurous odor of NAC precluded a double blind study. The control group only received 10 ml normal saline via their nebulizers 3 times a day at 8 AM, 2 PM, and 9 PM. An informed consent was obtained from first-degree relatives of the patients. Neither case nor control patients received other types of mucolytics. All patients in both groups were well hydrated, having an acceptable urine output and a good skin turgor. Suctioning of the ETT was done after each episode and secretions were gathered in a numerated bottle. Saline was not used for suctioning and the tip of suction tube was placed in the trachea just above the carina. Suctioning was done evenly in both groups without bias.

We placed a mucous extractor between the suctioning tube and the main suction container in order to obtain a sample from respiratory secretions and calculate its density. The density of respiratory secretion was calculated by dividing the weight of secretion per gram to its volume per milliliter. One milliliter of the secretion was picked up by a 1 ml syringe and its weight was measured by A&D GR-300 digital scale.

We recorded plateau and peak inspiratory pressures, auto-PEEP, and O2 saturation. We also auscultated the patient’s lungs, looking for rhonchi. This auscultation was done in a supine position with the stethoscope placed in 6 different areas; 4 on the anterior chest and 2 on the lateral chest of each patient. In order to eliminate bias produced by examining pulmonary sounds, we purchased an electronic stethoscope provided by Littmann (model 3200) which recorded these sounds. These lung sounds were analyzed by a pulmonologist. 

In each group, measurements and exam were done prior to the first nebulization as baseline evidence and they were repeated 12 and 24 hours afterwards.

Variables such as age, sex, underlying disease(s), and vital signs were also gathered.

Considering power (1-β) set at 0.80 and α=0.05, and the effect size of 0.205 by using G Power software,^9^ we estimated the total sample size would be at least 40.

Data were gathered and analyzed using SPSS version 11.5. We compared the variables between groups by Mann-Whitney, Friedman, and Fisher exact test according to the type of variables. A repeated measures ANOVA was conducted to compare the effect of NAC on respiratory secretion density, O2 saturation, and peak/plateau airway pressure at the baseline, 12 and 24 hours later. P value <0.05 was considered statistically significant. 

## Results

We studied 40 patients (21 women and 19 men). The mean ages of participants were 59.7±22 and 50.6±21 years in the case and control groups, respectively (P=0.289). Apart from the mean arterial blood pressure, no statistical difference was detected between the baseline demographics in either group (table 1). The receiving FIO2 through the study in both groups did not differ statistically (P=0.758). Different underlying diseases, including chronic obstructive pulmonary disease, diabetes mellitus, ischemic heart diseases, and congestive heart failure have been distributed in both groups without any significant difference (table 1).

**Table 1:** Demographic and clinical characteristics of control (normal saline nebulization) and case (N-acetylcysteine nebulization) groups

	**Control (n=20)**	**Case (n=20)**	**95% Confidence interval of the difference**		**P value**
			**Lower**	**Upper**	
Age (years)	50.6±21	59.7±22	-5.01	23.1	0.289
Gender (M/F)	8/12	11/9	-15.6	45.6	0.527
Temperature (C)	37.2±0.9	37.1±0.8	-0.61	0.54	0.779
Pulse rate (per min)	99±17	90±17	-21	1.8	0.108
Mean blood pressure (mmHg)	94.5±14	86.9±11	-16.01	82.1	0.049
FiO2 (%)	44.5±13	49.5±25	-7.8	17.7	0.758
Underlying diseases					
COPD (%)	10	20	-11.9	31.9	0.661
IHD/CHF (%)	10	25	-8	38	0.182
DM (%)	5	10	-11.2	21.2	1.000
Mortality (%)	7 (35%)	10 (50%)	-22.3	52.3	0.523

Patients had different diagnosis on admission, including; pneumonia, poisoning (carbon monoxide and drugs), and sepsis, but no significant difference was seen between the groups (not shown in table). 

The in-hospital mortality rate was 50% and 35% in the case and control groups that did not differ statistically (P=0.523). The recorded breath sound of patients revealed that the frequency of rhonchi within the control and case groups decreased significantly throughout the study (P=0.045 and P=0.002, respectively) (table 2).

**Table 2:** Mean and standard deviation of secretion density, plateau and peak airway pressures, and O2 saturation within control (normal saline nebulization) and case (NAC nebulization) groups at baseline versus 12 hours later and 24 hours later

**Variables**	**Baseline**	**12 hrs later**	**24 hrs later**	**P value**
Secretion density* (g/ml)				
Control	1.041±0.04	1.037±0.03	1.034±0.03	0.568
Case	1.021±0.03	1.011±0.02	1.001±0.02	0.065
Peak airway pressure* (cm H2O)				
Control	22.3±8.3	24.7±11.7	24.6±11.8	0.093
Case	27.1±11.8	26.5±11.8	26.0±11.2	0.303
Plateau airway pressure* (cm H2O)				
Control	19.3±9.3	21.2±11.4	21.2±9.6	0.131
Case	24.6±8.7	24.2±9.4	23.2±9.8	0.222
Peak plateau difference* (cm H2O)				
Control	6.3±4.8	8.3±6.9	8.8±7.4	0.184
Case	9.1±6.0	9.8±6.3	10±6.2	0.889
O2 Saturation* (%)				
Control	94.0±2.2	93.9±2.5	94.0±2.8	0.967
Case †	93.8±2.7	95.1±2.6	95.4±2.4	0.001
Rhonchi** (%)				
Control	50%	45%	20%	0.045
Case†	70%	40%	25%	0.002

*By repeated measure ANOVA; **By Friedman test; †Statistically significant

After reducing the dose of nebulized NAC from 3 to 2 ml of 20% NAC, we found no significant objective adverse effects of NAC such as bronchospasm, fever or rhinorrhea. As our patients were sedated, we could not evaluate other adverse effects of NAC such as drowsiness, clamminess, and chest tightness.

Table 2 shows the mean and the standard deviation of secretion density, plateau and peak airway pressures, and O2 saturation within the control (normal saline nebulization) and the case (NAC nebulization) groups at baseline versus 12 hours later and 24 hours later.

Although the mean secretion density was significantly lower in the NAC group (F (1,38)=8.61, P=0.006), but a repeated measures ANOVA with a Greenhouse-Geisser correction determined that the effect of NAC on the mean secretion density did not differ significantly between time points (F (1, 38)=3.08, P=0.087) (table 3).

**Table 3:** Source table for 3 (baseline, 12 hours, and 24 hours) x 2 (case- control) within subjects, within-between interaction, and between subject ANOVA of the study

**Sources**	**Sum of Squares**	**Degree of freedom**	**Mean square**	**F**	Sig.*
Secretion density	0.004	1	0.004	3.08	0.087
Density×group	0.001	1	0.001	0.82	0.372
Error (density)	0.046	38	0.001		
Between group (density)	0.021	1	0.021	8.61	0.006
Peak airway pressure	16.4	1.52	10.8	0.91	0.384
Peak×group	67.9	1.52	44.6	3.77	0.040
Error (peak)	666	56.4	11.8		
Between group (peak)	211	1	211	0.58	0.449
Plateau airway pressure	6.2	1.95	3.2	0.67	0.513
Plateau×group	30.2	1.95	15.4	3.29	0.049
Error (plateau)	174	37.1	4.69		
Between group (plateau)	187	1	187	0.67	0.423
Oxygen saturation	14.8	1.92	7.70	4.60	0.014
Saturation×group	16.2	1.92	8.42	5.02	0.010
Error (saturation)	122	73.1	1.67		
Between group (saturation)	18.4	1	18.4	1.09	0.304

*Calculated Based on Greenhouse-Geisser correction

**Figure 2:**
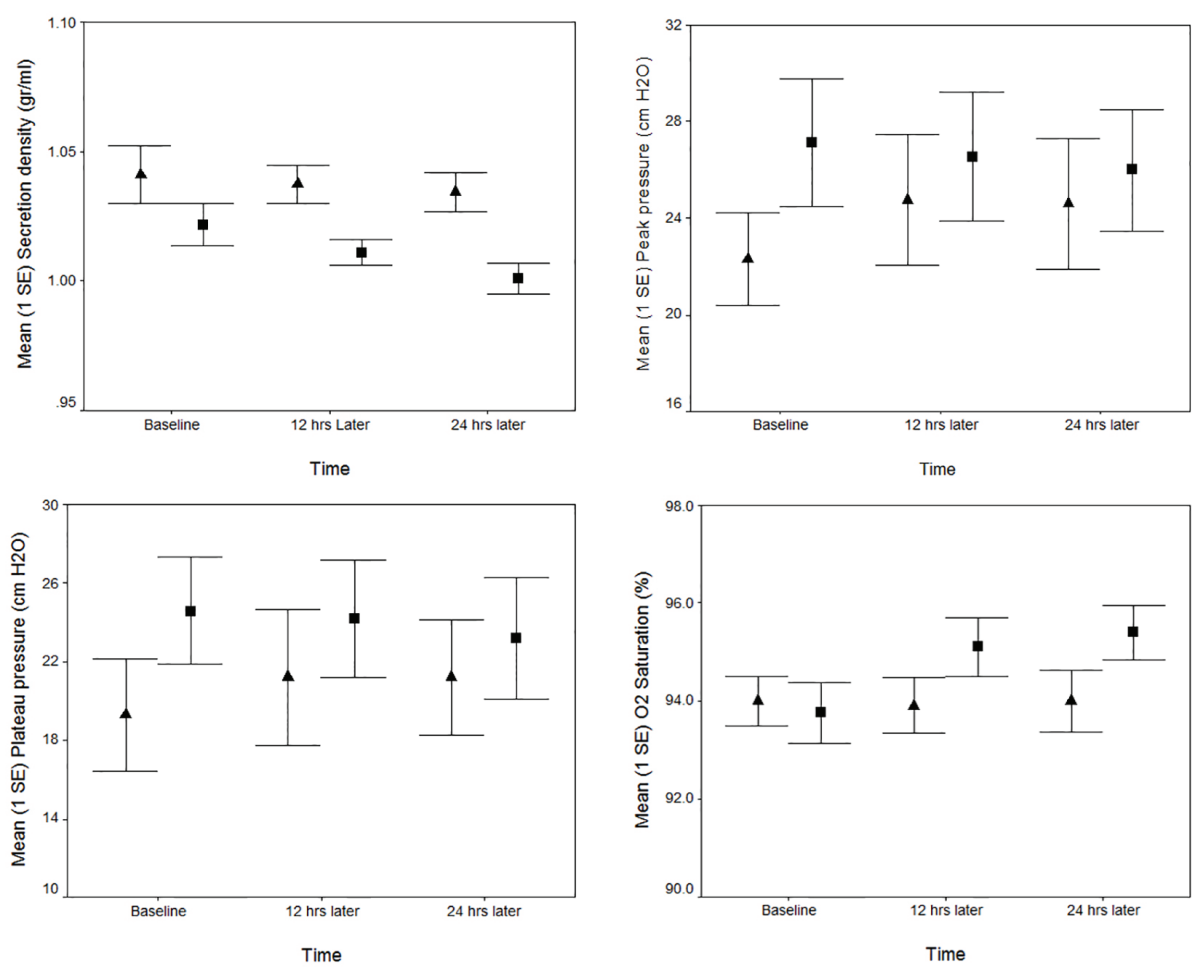
The mean (SE) of secretion density, plateau and peak airway pressures, and O2 saturation in control (normal saline nebulization) and case (N-acetylcysteine nebulization) groups at baseline, 12 and 24 hours later (▲ for controls and ■ for cases).

The plateau airway pressures were relatively stable throughout the study in the normal saline and NAC groups (F (1.95, 37.1)=0.67, P=0.513) (figure 2). The peak airway pressure did not change significantly during the study in the normal saline and NAC groups (F (1.52, 56.4)=0.91, P=0.384) (figure 2). The differences between peak and plateau airway pressures were relatively stable throughout the study in both groups (table 2).

Analysis revealed NAC significantly increased O2 saturation between time points (F (1.92, 73.1)=4.6, P=0.014). There was a significant interaction between treatment and time, F (1.92, 73.1)=5.02, P=0.01 (table 3). This effect indicated that the rise in saturation significantly differed in the NAC and normal saline groups. While during the study we had not changed the FIO2 of ventilator in both groups, Bonferroni corrected post hoc tests showed that 12 hours after starting the study, the oxygen saturation in the case group had a statistically significant increase from 93.75±2.7 to 95.10±2.6 (P=0.001) and then reached a plateau during the terminal 12 hours (P=0.902).

## Discussion

This study demonstrated that using N-acetylcysteine via nebulization through endotracheal tubes in mechanically ventilated patients was not effective more than normal saline nebulization in reducing the density of mucous secretions. A significant increase in the trend of oxygen saturation was seen in the group who received nebulization of NAC.

Poppe reported a personal experience regarding instillation and inhalation of 2 ml NAC 20% in 88 patients. These patients showed improvement in clinical signs and symptoms. The study showed a more dramatic effect following instillation of this mucolytic compared with its aerosol inhalation. He found that the instillation of NAC could induce prolonged series of cough for about ten minutes.^10^

In Hirsch’s study, the effect of one episode nebulization of 3 ml NAC 20% and 3 ml NAC 10% were compared with normal saline nebulization. They concluded that nebulized NAC was more effective than normal saline nebulization in thinning sputum and increasing its volume and that 10% NAC was as effective as 20%.^11^ Kory et al.^12^ and Hirsch et al.^13^ showed that nebulized NAC plus bronchodilators such as racemic epinephrine and isoproterenol were not only highly effective in thinning sputum but also were associated with subjective improvement that could be related to the use of bronchodilators.

In the above mentioned studies, NAC 20% and NAC 10% were used and the consistency of sputum was measured using a consistometer; while we used 30 ml NAC 4% (three doses of 2 ml NAC 20% diluted in 8 ml normal saline) divided in three doses and we measured the density of sputum.

As a matter of fact, density and viscosity are two different physical properties of fluids; nevertheless we expected to find a statistically significant decline in the density of respiratory secretions, which we did not. It might be explained by the lower amount of NAC per dose or relatively short period of our study. On the other hand, by reducing the amount of NAC per dose even without adding a bronchodilator, none of our patients experienced significant NAC-induced bronchospasm. 

The increment in oxygen saturation in this study is in agreement with Gallon’s study.^14^ In Gallon’s study, the improvement in oxygen saturation after nebulization of NAC was associated with a reduction in sputum viscosity, increase in sputum expectorated weight, and easier expectoration of sputum.^14^ In our study, the statistically significant increment in oxygen saturation of NAC group was not accompanied by any significant detriments in the density of respiratory secretions or airway pressures. Thus, it cannot be explained by mechanical changes in airways. This finding could be explained by improved mucociliary clearance after nebulizing NAC^15^ or fluctuation in oxygen saturation during the day as shown by Vargas^16^^,^^17^ Vargas et al. found that, oxygen saturation has fluctuation with maximum values in the late afternoon and minimum values in the early morning.^16^^,^^17^ Agusti et al. studied patients with multiple organ failure and showed a significant decrement in oxygen saturation 45 minutes after starting IV NAC.^18^ Such disagreement between our study and Agusti’s study could be due to differences in study population, route of administration of NAC, and the timing of oxygen saturation measurement.

Our trial studied 20 cases receiving three episodes of NAC nebulization for a period of 24 hours using 2 ml of NAC in each episode. 

To check whether our non-significant results were due to a lack of statistical power, we conducted power analyses using G Power^9^ with power (1-β) set at 0.80 and α=05, and related effect size calculating from partial Eta squared. In order to reach statistical significance at the 0.05 level for secretion density differences, we found that sample sizes would have to be 22, 26, and 78 for within group, between groups, and within-between interactions, respectively. Thus, except for detecting within-between interaction respiratory secretion density differences, it is unlikely that our negative findings regarding within and between groups can be attributed to a limited sample size.

Our study had limitations. We were unable to measure viscosity of the secretions since a rheometer was unavailable. The second limitation was the relatively short period of our study. 

## Conclusion

Considering the limitations of the study, we concluded N-acetylcysteine via nebulization through endotracheal tubes in mechanically ventilated patients was not effective more than normal saline nebulization in reducing the density of mucous plugs. The peak and plateau airway pressures were relatively stable throughout the study in both groups. 
